# Juvenile idiopathic arthritis in infants with Harlequin Ichthyosis: two cases report and literature review

**DOI:** 10.1186/s13052-020-0817-5

**Published:** 2020-04-15

**Authors:** Cinzia Auriti, Roberta Rotunno, Andrea Diociaiuti, Silvia Magni Manzoni, Andrea Uva, Iliana Bersani, Alessandra Santisi, Andrea Dotta, May El Hachem

**Affiliations:** 1 Department of Medical and Surgical Neonatology, Bambino Gesù Children’s Hospital, IRCSS, Piazza S. Onofrio 4, 00165 Rome, Italy; 2 Pediatric Dermatology Unit, Bambino Gesù Children’s Hospital, IRCCS, Rome, Italy; 3 Rheumatology Division, Bambino Gesù Children’s Hospital, IRCCS, Rome, Italy; 4 Pediatrics Department, Umberto I Hospital, La Sapienza University, Rome, Italy

**Keywords:** Harlequin Ichthyosis, Juvenile idiopathic arthritis, Oral retinoids, Acitretin, Case report

## Abstract

**Background:**

Harlequin Ichthyosis is the most severe variant of congenital autosomal recessive ichthyosis, associated with severe morbidity and potentially lethal in early life. At birth, patients present thick and plaque-like scales all over the body, with consequent cutaneous and extra-cutaneous complications, such as poor thermoregulation, recurrent infections, pain, electrolytes imbalance and joint contractures. Juvenile Idiopathic Arthritis usually manifests before the age of 16 years and persists for more than 6 weeks. The association between these two pathologies has been described in the literature as a very rare event, which creates diagnostic and therapeutic challenge.

**Case presentation:**

We describe two patients affected by Harlequin Ichthyosis who early developed Juvenile Idiopathic Arthritis. Both patients were treated with retinoids, ibuprofen and long-acting intra-articular glucocorticoids; due to polyarticular involvement, one child was also treated with weekly oral methotrexate.

**Conclusions:**

The association between Harlequin Ichthyosis and Juvenile Idiopathic Arthritis is rare and the pathophysiological mechanism that binds them is still unknown. Nonetheless caregivers should be aware of the possible occurrence of Juvenile Idiopathic Arthritis at very early ages in children affected by Harlequin Ichthyosis.

## Background

Harlequin Ichthyosis (HI) is a severe genetic disorder of skin keratinization, caused by mutations in the ABCA12 gene, inherited as autosomal recessive trait. The estimated incidence is less than 1/300.000 births [1]. HI represents the most severe form of non-syndromic ichthyoses, associated with perinatal morbidity and potential lethality early in the life [1, 2]. At birth, patients present thick and plaque-like scales all over the body, ectropion, eclabium and ears deformities, evolving in severe desquamative erythroderma. Poor thermoregulation, recurrent infections, poor feeding, pain, imbalance electrolytes, and joint contractures are the most frequent complications. Juvenile Idiopathic Arthritis (JIA) is the most common rheumatic disease of childhood causing disability and blindness. By definition JIA encompasses all forms of arthritis that begin before the age of 16 years, persist for more than 6 weeks, and are of unknown cause, with a reported overall incidence of 15–150/100.000 [3].

We describe two patients affected by HI, who developed a persistent inflammatory arthritis, consistent with JIA, in the first 2 years of life. This combination has been rarely reported in the literature, never during the infancy. The pathogenic association of the two conditions is still unknown.

Parental consents were obtained for publication of the clinical histories of the children and pictures.

## Cases presentation

### Patient 1

**Fig. 1:**
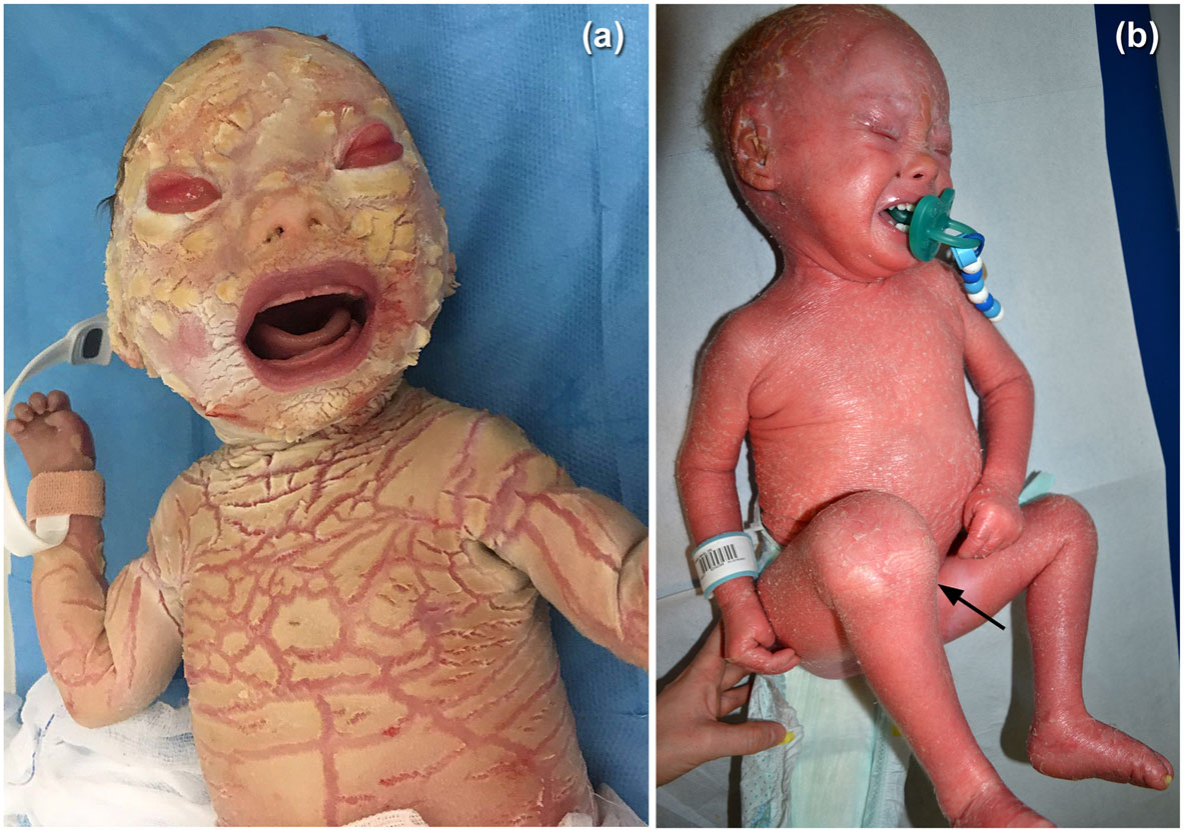
Patient 1: Clinical presentation at birth **a**; Swelling of the right knee **b**, mimicking a septic arthritis, at 3 months of life

A 2.300 g boy was born at 35 weeks, by cesarean section due to Premature Rupture Of the Membranes (PROM) Parents were not related and pregnancy was uneventful, except for gestational diabetes requiring insulin. At birth, the neonate showed thickened skin, opaque plate-like keratotic scales punctuated by deep red fissures, ectropion, eclabium, flattened nose, dysmorphisms of ear cartilage and auditory ducts ending in the neck. Fingers and toes presented flexion contractures, strictures and pseudo-syndactyly (Fig. 1 a). The neonate was admitted in our Neonatal Intensive Care Unit, breathing spontaneously and stable from the cardio-respiratory point of view. Since the first day we started oral retinoids (1.5 mg/Kg of acitretin into two divided doses). In addition, the baby underwent global care to improve skin hydration and to prevent and/or reduce extracutaneous complications. The skin treatment included ointments containing glycerin, paraffin oil and vaseline, up to six times a day. Eosine 2% was applied on skin fissures. Artificial tears and subsequent medication with sterile gauzes soaked in isotonic solution were applied on eyes four times a day. Vaseline was applied on scalp and eyebrows as well. Genetic testing confirmed the clinical suspicion of HI, identifying a compound heterozygosity c.4036delG and c.7444C > T genomic variants in the *ABCA12* gene. Blood levels of vitamin D, parathyroid hormone, alkaline phosphatase, electrolytes, hepatic and renal function and complete blood count were within normal ranges. During hospitalization the baby underwent multiple red cells and platelets transfusions for anemia and thrombocytopenia and several intravenous antibiotics for recurrent sepsis. At 2 months we detected severe bilateral transmissive deafness, due to otodisplasia. At 3 months the infant showed a marked swelling of the right knee, mimicking a septic arthritis and therefore initially treated with systemic antibiotics, without complete resolution of knee swelling (Fig. 1 b). During the following months recurrent impairment of knee arthritis occurred, with mild increase in C-Reactive Protein (CRP) and erythrocyte sedimentation rate (ESR), while antinuclear antibodies as well as rheumatoid factor (RF) were negative. Ultrasound of the knee showed clear effusion in the right supra-rotuleal bursa (27 × 9.5 × 30 mm) associated with synovial thickening and increased vascularization. Oral non-steroidal anti-inflammatory drug (NSAID) was administered without improvement of symptoms. At 2 years arthrocentesis with aspiration of yellow turbid synovial fluid and subsequent intra-articular injection of triamcinolone hexacetonide (1 mg/kg) were performed, with immediate relief. Additional oral NSAID course and physiotherapist rehabilitation program further improved symptoms. On year later, complete remission of the arthritis of the right knee was obtained without any limitation or side effects; no other joints have been involved in the meantime. Acitretin was progressively tapered, due to the improvement of the skin conditions, in particular eclabium and ectropion, and stopped at 10 months of age.

### Patient 2

**Fig. 2:**
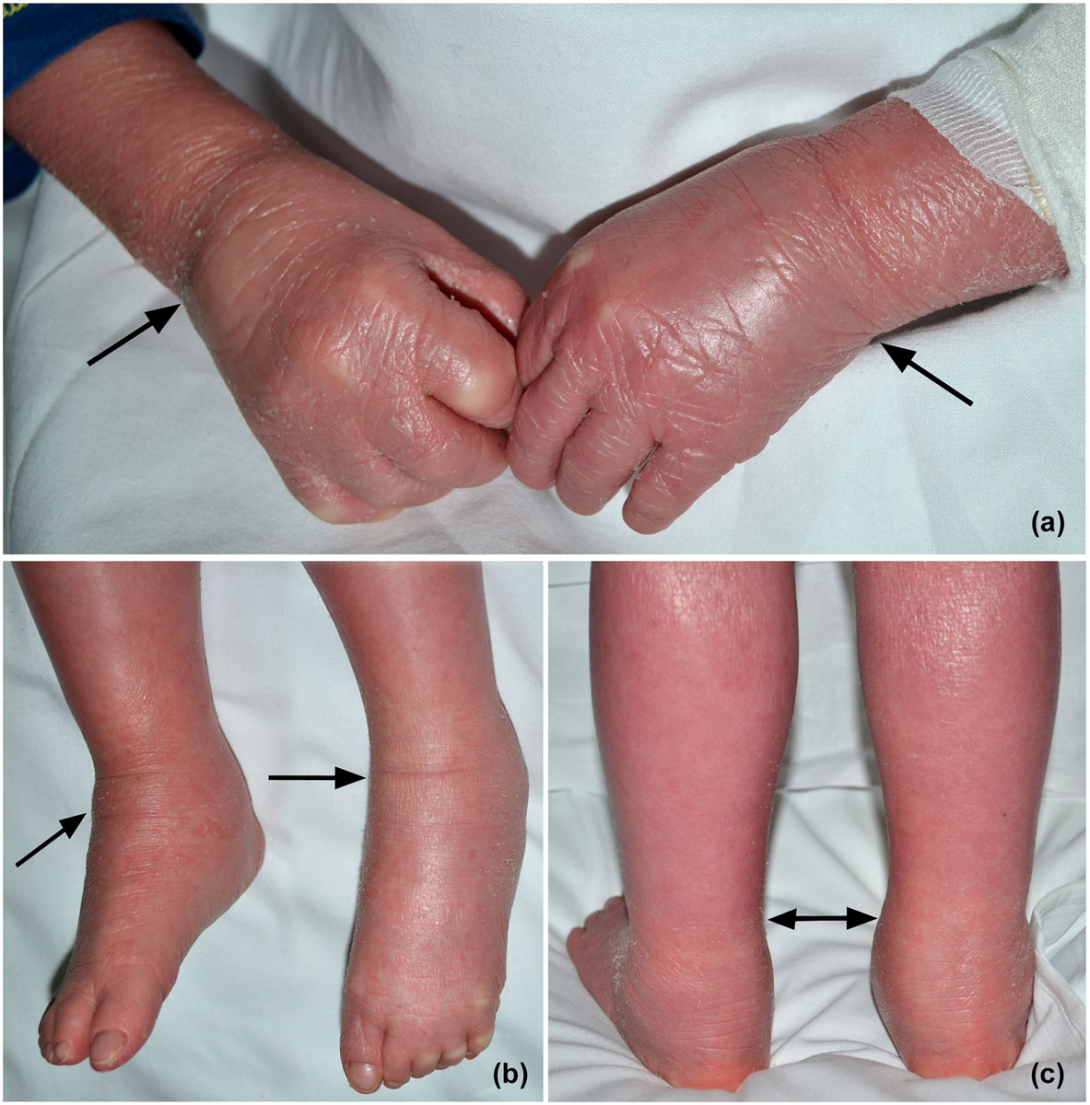
**Patient 2** Swelling of the hands **a** and ankles **b** and **c** at 2 years of life

A 3.048 g. male, single child of healthy not related parents, was delivered at 36 weeks and 5 days via cesarean section. At birth the baby presented persistent bradycardia (heart rate < 60 bpm), hypotonia and generalized cyanosis, underwent 30 s cardio-pulmonary resuscitation and cried at 4 min, with recovery of cardiorespiratory stability. His whole body was covered by collodion-like membranes, thicker in the scalp, lower limbs and upper arms were flexed, contracted and covered by a thickened skin with multiple fissures. He presented also ectropion and deformity of the auricles. In the suspicion of congenital ichthyosis, the baby was referred to our Unit, where a skin biopsy and a molecular testing were performed. Moreover the baby presented sepsis due to *Enterococcus faecalis* and bronchitis, requiring antibiotic therapy, since the admission to our Unit. Histological and genetic exams confirmed respectively the diagnosis of autosomal recessive congenital ichthyosis, sustained by the genomic variants c.224 T > A, c.6610C > T, c.164G > A and c.346G > T in heterozygosity composed in the *ABCA12* gene and the variant c.817G > A in heterozygosity in the *TGM1* gene. Such mutations in *ABCA12* determine the p. Leu75His (rs147793298), p. Arg2204Ter (rs137853289), p. Cys55Tyr, p. Asp116Tyr (rs141700130) and p. Gly273Arg variants, respectively, at a protein level. The p. Leu75His e p. Arg2204Ter variants were due to paternal segregation. We promptly started oral therapy with acitretin (1.5 mg/kg/day). The improvement of skin conditions, ectropion, ears deformity and eclabium was remarkable, but after 1 month we had to stop therapy because the increase of liver enzymes; in any case the physiotherapist rehabilitation program started during the hospitalization lead to a significant improvement of the patient’s musculoskeletal mobility. After the discharge the baby manifested recurrent respiratory infections, which required multiple courses of antibiotics and glucocorticoids. At 2 years he developed painful swelling of the hands, left knee, both elbows, wrists and ankles (Fig. 2a, b and c). The rheumatologist’s assessment revealed polyarthritis of both elbows and wrists, right knee, left tibiotalar, subtalar and talonavicular joints, associated with diffuse tenosynovitis at several flexor tendons of the fingers, extensors of the wrists, both extensors and flexors of the ankles, as confirmed by musculoskeletal ultrasound examination. The child could not walk or even stand without the parents’ help. Laboratory tests showed the increase of WBC, eosinophils, ESR, CRP, IgE, with negative RF and low levels of vitamin D. Transient relief was initially provided by short oral ibuprofen courses. Nonetheless, due to the worsening of symptoms at every ibuprofen discontinuation, we decide to start local therapy with long-acting glucocorticoid intra-articular injections (triamcinolone hexacetonide, 20 mg/mL and acetate methylprednisolone, 40 mg/ml) in large and small joints or tendon sheaths, under ultrasound-guidance and sedation. We started also systemic therapy with oral methotrexate (15 mg/mq/weekly) and folinic acid (7.5 mg/mq/weekly), with remarkable improvement of symptoms, complete pain relief and return to daily usual activities. After 2 months from the start of the therapy we documented the complete clinical and ultrasound remission in all the injected sites, except the persistence of both ankle swelling, due to diffuse tenosynovitis of the anterior and lateral compartments. To date the child is still under follow-up, an additional injection procedure and methotrexate administration have been planned in the early future. The baby did not manifest side effects related to both treatments.

## Discussion and conclusions

### Discussion

Harlequin Ichtyosis is an autosomal recessive genetic disorder due to the mutation of the *ABCA12* gene in the chromosomal region 2q35. This gene belongs to a superfamily of ATP-binding cassettes, involved in the transport of biomolecules across the cell membrane, in the differentiation of keratinocytes and in the formation of the epidermal lipid barrier [4–8]. Mutations are classified as homozygous (same mutation in both alleles) or compound heterozygous (different mutations on each allele) [9]. Ominous outcome is usually associated with homozygous mutations. Our patients were carriers of a compound heterozygosity. Parents transmit the pathology to 25% of their children: they transmit the mutation of the ABCA12 gene in duplicate, at each conception, regardless of the sex of the unborn child; half of the children can be healthy carriers (heterozygous for the mutation of the ABCA12 gene, meaning having a single mutation of the disease gene), 25% healthy. However, some children with HI show no mutations of the ABCA12 gene, suggesting that the genetic basis underlying HI is still far from to be clear [9]. In HI babies, skin is markedly thickened, the hard stratum corneum cracks soon after birth and leads to deep erythematous fissures delimiting geometric skin plates, with alopecia, ectropion, eclabium, and flattened ears. Dehydration, hindered thermoregulation, increased metabolic demands, feeding disorders, respiratory distress, fingers fixed in flexion by tight skin and increased risk of infection may occur [1, 10]. Skin of survivors evolves to generalized erythema and scaling, often with associated palmoplantar keratoderma. Developmental delay is frequently described as well [9]. The first line therapy of HI is oral retinoids and should be started early as much as possible [11, 12]. The use of incubators with 50–85% humidification, the skin care with emollients and advanced dressing for fissures, eyes care with artificial tears and the close monitoring of possible extracutaneous complications are strongly recommended [1, 9, 11, 12]. In some patients the surgical debridement of the joints of the hands and feet is inevitable, to allow movement and to avoid finger/toe’s necrosis. Concerning the drug therapy, acitretin at the dose of 0.5–1 mg/kg/day is the most used among different retinoids in neonates, thanks to less severe side effects compared to other compounds [13, 14]. Retinoid administration may be discontinued by 6 months of age [11, 12]. It is still unclear whether the use of oral synthetic derivatives of vitamin A in neonates is associated with bones demineralization and/or fractures, as suggested by some authors. Only few case reports exist [1, 15]. Sitek et al. reported the case of an infant affected by HI treated with oral acitretin who developed osteopenia and multiple diaphyseal fractures of the four limbs [16]. In both our patients acitretin was successful and interrupted after few months, because the improvement of facial deformities and the skin thickening, without bone demineralization and/ or fractures. However, our infants developed a precocious JIA, a pediatric chronic inflammatory disease, rarely reported in HI patients so early in the life. Chan et al. reported the case of a 11-year-old Chinese girl affected by erosive arthritis with rheumatoid factor-positive, diagnosed as polyarticular JIA at 6 years. The child was successfully treated with prednisolone and NSAIDs up to the age of 9 and then with methotrexate at the dose of 0,5 mg/Kg/week also, because the poor control of symptoms with prednisolone alone [17]. The clinical improvement of erythema and arthritis was not associated with the parallel improvement of the hyperkeratosis and scaling. The child underwent etretinate therapy from 36 days to 6 years, when the drug was replaced by acitretin. Authors conclude the report warned about erosive arthritis, as an adverse event associated with etretinate therapy. To date this side effect of etretinate administration is not reported in the literature. Our patients were treated for a short time (10 months and 1 month for patients 1 and 2 respectively) and we think that acitretin may not have been the cause of JIA in them. The first line therapy for JIA was started with ibuprofen. Both patients underwent intra-articular long-acting glucocorticoid injections, with additional weekly oral methotrexate administered to the patient 2, as in the 6 years old patient reported by Carbogno et. Al [18]. Clement et al. reported a 14-year-old boy, who suffered from both HI and JIA, managed with traditional NSAIDs and methylsulphone COX-2 inhibitors [19]. Authors recommended to adequately treat JIA, because the because the reduction of mobility worsens contracture and joint swelling, accentuates skin lesions and increases the risk of infection. Another 17-years-old case, who developed JIA at the age of 10, underwent bilateral total hip replacement, after initial treatment with etoricoxib and etanercept [20].

The relationship between JIA, HI and oral retinoid therapy has not been yet elucidated. Although *ARBCA12* gene is not associated with known immunologic cells defects, it has been proved that its mutations lead to an impairment of the outermost layer of the skin, which is the first mechanism of defence against microbes (especially Gram-positive bacteria) capable of causing disseminated infections. JIA related risk has been associated both to infections during the first year of life [21] and to the extensive use of antibiotics early in the life [22]. With regards to this hypothesis, it has been supposed that the antibiotic treatment could cause JIA though changes in microbiome [23]. Of note, disbyosis in children with JIA has been documented [24] and it is not of univocal interpretation, as it also could be the expression of the inflammation derangement status, linked to the chronicity of the disease. Our patients may support this hypothesis, because they both presented recurrent infections treated with antibiotics. It may be of interest testing HI patients with arthritis for described JIA genetic association (i.e. HLA polymorphism). To date our patient 1 has gone to complete and persistent remission of his monoarticular involvement at one-year evaluation. The patient 2, despite the start of methotrexate therapy, presents persistent tenosynovitis of both ankles at 2 months evaluation, while joints and tendons of the hands, treated with glucocorticoids injections, have undergone clinical and ultrasound remission. Periodic follow-up and treatment adjustments have been planned in both children.

### Conclusions

HI has been considered a fatal disease for decades and the clinical course has currently improved thanks to the multidisciplinary approach [1, 25, 26]. HI neonates should be managed in neonatal intensive care units, starting short-course therapy with oral retinoids early and preventing infections with special attention. Systematic efficacy/safety studies, that allows to evaluate the most appropriate therapeutic scheme, rather than single reports, are however strongly required.

The possible development of JIA already in the early age of life should be always be kept in mind by caregivers and our experience suggests that the association between HI and JIA could be related to some inflammatory relationships between joints and skin and offer new research insights.

## Data Availability

Data supporting the results reported in the article can be found in the intranet of Bambino Gesù Childrens’ Hospital.

## Abbreviations

CRP: C Reactive Protein
ESR: Erythrocyte Sedimentation Rate
HI: Harlequin Ichthyosis
JIA: Juvenile Idiopathic Arthritis
NSAID: Non-steroidal Anti-Inflammatory Drug
PROM: Premature Rupture Of the Membranes
RF: Rheumatoid Factor
WBC: White Blood Cells
